# Large-area high-performance SERS substrates with deep controllable sub-10-nm gap structure fabricated by depositing Au film on the cicada wing

**DOI:** 10.1186/1556-276X-8-437

**Published:** 2013-10-22

**Authors:** Qi Jiwei, Li Yudong, Yang Ming, Wu Qiang, Chen Zongqiang, Wang Wudeng, Lu Wenqiang, Yu Xuanyi, Xu Jingjun, Sun Qian

**Affiliations:** 1 The MOE Key Laboratory of Weak-Light Nonlinear Photonics, Tianjin Key Laboratory of Photonics and Technology, TEDA Applied Physics School and School of Physics, Nankai University, Tianjin 300457, China

**Keywords:** Surface-enhanced Raman scattering, Controllable gap size, Cicada wing, Nanogap

## Abstract

Noble metal nanogap structure supports strong surface-enhanced Raman scattering (SERS) which can be used to detect single molecules. However, the lack of reproducible fabrication techniques with nanometer-level control over the gap size has limited practical applications. In this letter, by depositing the Au film onto the cicada wing, we engineer the ordered array of nanopillar structures on the wing to form large-area high-performance SERS substrates. Through the control of the thickness of the Au film deposited onto the cicada wing, the gap sizes between neighboring nanopillars are fine defined. SERS substrates with sub-10-nm gap sizes are obtained, which have the highest average Raman enhancement factor (EF) larger than 2 × 10^8^, about 40 times as large as that of commercial Klarite® substrates. The cicada wings used as templates are natural and environment-friendly. The depositing method is low cost and high throughput so that our large-area high-performance SERS substrates have great advantage for chemical/biological sensing applications.

## Background

Surface-enhanced Raman scattering (SERS), as a powerful spectroscopy technique that can provide non-destructive and ultra-sensitive characterization down to a single molecular level [1,2], is currently receiving a great deal of attention from researchers. Lots of works focus on the SERS mechanism and the fabrication of high-performance SERS-active substrates for application [3-44]. High-performance SERS substrates mean that the substrates should be uniform, reproducible, and ultra-sensitive.

Recently, the nanogap structure becomes attractive to researchers because it can provide enormous Raman enhancement due to the existence of enormous electromagnetic enhancement in the gap of metal nanostructure, which is called 'hot spot’ [3-16]. And the surface plasmonic coupling between neighboring nanounits is believed to be the main reason for the enormous electromagnetic enhancement. Many investigations on the mechanism of the surface plasmonic coupling and the fabrication of the nanogap-structured SERS substrates for practical application have been presented [3-17]. Compared to the nanoparticle substrates, the ordered nanopillar/nanorod array substrates are more uniform and reproducible, which make them more beneficial to practical application and theoretical analysis. But the uniform ordered nanopillar/nanorod array substrates with tunable gap size are usually fabricated by electron-beam lithography (EBL) and focused ion-beam lithography (FIBL), which require a very high fabrication cost [18-20]. To circumvent this difficulty, many low-cost methods and techniques have been proposed, like self-assembly [21,22], indentation lithography [14,20,23-27], corroding ultra-thin layer [7], femto-second laser fabrication [28-31], and so on. But to date, for the existence of many limits of these low-cost techniques, the fabrication of the large-area low-cost high-performance SERS substrate, with tunable gap size, is still critical not only for practical applications of SERS in the chemical/biological sensor, but also in understanding surface plasmonic coupling existing inside the nanogaps.

In this letter, we provide a simple method to fabricate large-area low-cost high-performance SERS substrates with tunable gap size through depositing the Au film onto the ordered nanopillars array structure on the cicada wings. The fine control of the gap size is achieved by controlling the Au film deposition thickness. The dependence of the average enhancement factor (EF) on the gap size is investigated. The highest average EF, 2 × 10^8^, is obtained when the gap size is <10 nm. This highest average EF is about 40 times as large as that of commercial Klarite® substrates. The large-area low-cost high-performance SERS substrates with tunable gap size, obtained in our work, not only are useful for improving the fundamental understanding of SERS phenomena, but also facilitate the use of SERS for chemical/biological sensing applications with extremely high sensitivity. In addition, because the cicada wings used as the templates in our work are from nature, our SERS substrates are environment-friendly.

## Methods

### Sample and substrate preparation

**Figure 1:**
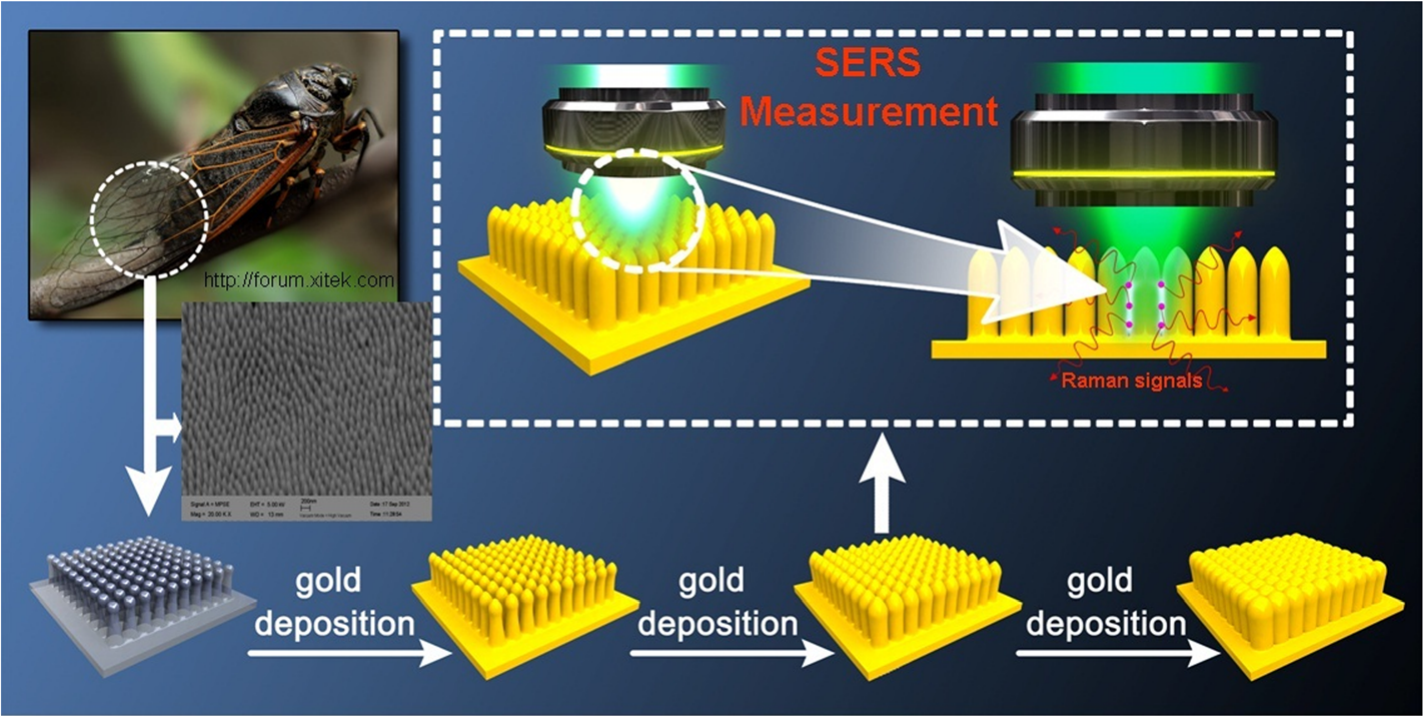
**Schematic illustration of the fabrication program of the SERS substrates.** The ordered array of nanopillar structures on the cicada wing was used directly as the template. The SEM image and schematic illustration of the nanopillar structures are shown. The Au films were deposited on the cicada wings to engineer the nanostructures and define the gap size.

Many nanostructures existing in biology are evolutionary results for the needs of adaptation and survival, which can produce astonishing optical effects and can be used directly. An ordered array of nanopillar structures on the cicada wing, with a perfect anti-reflection efficiency, has been investigated widely [45-48] and was used as the template in this letter. The cicadas (*Cryptympana atrata* Fabricius) were captured locally. Before the depositing process, the cicada wings are cleaned to get rid of the stains and restored the sticking nanopillars. Figure 1 shows the scanning electron microscope (SEM) image of the cicada wing and schematic illustrations of the fabrication of the SERS substrates. A hexagonally quasi-two-dimensional (q2D) ordered assembly of nanopillars exists on the surface of the cicada wing. The nearest-neighbor nanopillar distance (*Λ*) is an approximate 190 nm; the average height (*h*) of each nanopillar is about 400 nm, and the average diameter at the pillar top and base are about 65 and 150 nm, respectively. The main component of the cicada wing is chitin - a high molecular weight crystalline polymer [47]. And due to the existing of the ordered array of nanopillars, the cicada wing shows an excellent anti-reflection [46-48]. Here, the cicada wing, with a large-area uniform nanostructure on the surface, was used as the template. As shown in Figure 1, the Au film was deposited onto the surface of the cicada wing with an ion beam sputter evaporator to engineer the nanostructure. The Au film thicknesses (*d*) were controlled to be 50, 100, 150, 200, 250, 300, 350, and 400 nm, respectively, and these SERS substrates were signed with CW50, CW100, and so on in the following discussion. The deposition process was kept with target substrate at room temperature with a depositing rate of 0.03 nm/s.

**Figure 2:**
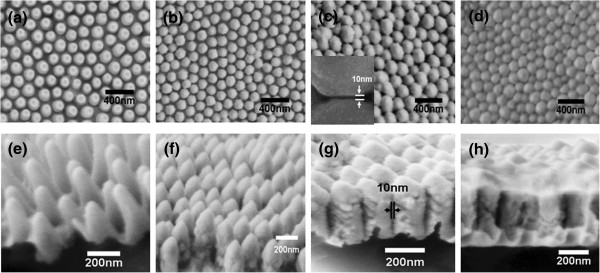
Top-(a,b,c,d) and side-view (e,f,g,h) SEM images of SERS substrates CW50, CW200, CW300, and CW400, respectively.

**Figure 3:**
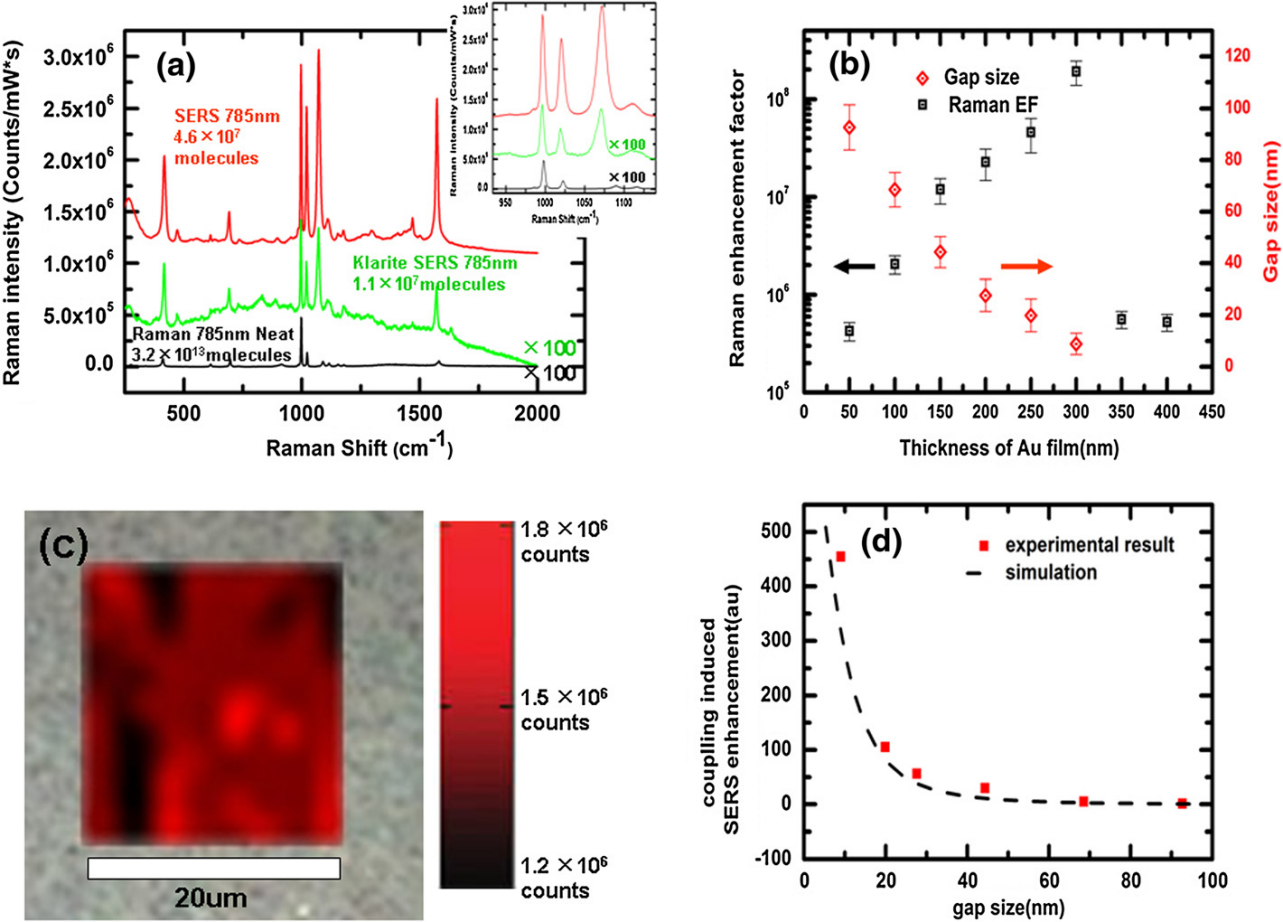
**Comparison of substrates and neat benzene thiol, average EFs and gap sizes, spatial mapping, and COMSOL simulations. (a)** Comparison of the SERS of substrates CW300 (red), Klarite® (green), and neat Raman spectra (black) of benzene thiol collected at 785-nm incident. The number of molecules of benzene thiol that each measurement is probing is denoted in the figure. Inset: zoomed-in region of the spectra showing the three primary modes located near 1,000/cm, with the 998/cm used for calculation of the SERS enhancement factor. Note that the SERS of the Klarite® substrate and the neat spectra have been multiplied by a factor of 100 for easier direct comparison. **(b)** Average EFs (black open squares) and gap sizes between neighboring nanopillars (red open rhombuses) as function of gold film thickness deposited on the cicada wing. **(c)** Spatial mapping of the SERS intensity at 998/cm of SERS substrate CW300 over an area larger than 20 μm × 20 μm. The background is the optical reflection image of substrate CW300 photographed through a microscope with a × 50 objective. **(d)** COMSOL simulations of SERS enhancement (black dash) and the mean of experimental average EFs (red squares) as function of gap size between neighboring nanopillars. All date points are normalized to the corresponding value of SERS enhancement of CW50.

Figure 2a,b,c,d and Figure 2e,f,g,h show the top view and side view SEM images of CW50, CW200, CW300, and CW400, respectively. As shown in Figure 2, with the increase in the deposited Au film thickness *d*, when *d* ≤ 300 nm, the gap size (*g*) between the nearest-neighbor nanopillars decreases, and the nanopillars tend to become hexagonal nanorods. The average *g* of CW50 to CW300 were measured with commercial software and shown in Figure 3b. According to the measured results, the average *g* even decreases to sub-10 nm when *d* is 300 nm. The average heights of the nanopillars (*h*) of CW50 to CW300 were also measured, and the measurement results show that the average height of the nanopillars (*h*) decreases from about 400 nm to about 200 nm with the increase in *d*. This is reasonable because with the decrease of *g*, the gold atoms are easier to fall into the bottom which leads to a faster rise of the bottom. Additionally, the surfaces of the nanopillar structures of CW50, CW100, and CW150 are relatively smooth; contrarily, the surfaces of the nanopillar structures of CW200, CW250, and CW300 are relatively rough. When *d* > 350 nm, i.e., the cases of CW350 and CW400, relatively continuous layers formed on the top of the nanopillars. The relatively continuous layers are rugged and the nanopillars were covered up so the *g*s of CW350 and CW400 cannot be measured and shown in Figure 3b.

### SERS spectra measurement and EFs calculation

To characterize the SERS performance of our substrates, benzene thiol was used as the probe molecule. And commercial Klarite® substrates were used as reference samples. The Klarite® SERS substrate consists of a gold-coated textured silicon (regular arrays of inverted pyramids of 1.5-μm wide and 0.7-μm deep) mounted on a glass microscope slide. All of the substrates (including Klarite® substrates) were immersed in a 1 × 10^-3^ M solution of benzene thiol in ethanol for approximately 18 h and were subsequently rinsed with ethanol and dried with nitrogen to ensure that a complete self-assembled monolayer (SAM) was formed on the substrate surface. All the Raman spectra were recorded with a confocal Raman spectroscopic system (model inVia, Renishaw Hong Kong Ltd., Kowloon Bay, Hong Kong, China). The spectrograph uses 1,200 g/mm gratings, a 785-nm laser, and a SynchroScan type camera. The incident laser power for different SERS substrates were not the same because of the huge difference of the Raman sensitivity among the substrates. The incident laser power was set to be 0.5 mW for CW350 to CW400 and 0.1 mW for CW50 to CW100 and Klarite® substrates 0.05 mW for CW150 to CW200 and 0.005 mW for CW250 to CW300. All the SERS spectra were collected using a × 50, NA = 0.5, long working distance objective. The laser spot size is about 2 μm. SERS spectra were recorded with an accumulation time of 10 s and a single scan was performed after SAM of benzene thiol formed on the substrate surface. To get an accurate approximation of the enhancement factors, the neat Raman spectrum of benzene thiol was measured. For these measurements, the power of the 785 nm laser was 1 mW, the accumulation time was 10 s, the spot size was 20 μm, and the depth of focus was 18 μm. Figure 3a shows the Raman spectra of the benzene thiol SAM on the optimal substrate (CW300; red), Klarite® substrate (green), and neat thiophenol (black), with everything being normalized to account for the accumulation time and laser power. The number of molecules contributing to the Raman signal was quoted in Figure 3a and was used for calculating EFs. The average EFs were calculated from the equation

$$\mathrm{EF}=\left(I_{\mathrm{SERS}}/I_{\mathrm{Raman}}\right)\times \left(N_{\mathrm{Raman}}/N_{\mathrm{SERS}}\right),$$

where *I*_SERS_ and *I*_Raman_ represent the normalized Raman intensity of SERS spectra and neat Raman spectrum of benzene thiol, respectively, which can be measured directly from the Raman spectra. *N*_SERS_ and *N*_Raman_ represent the numbers of molecules contributing to SERS signals and neat Raman signals of benzene thiol, respectively. *N*_Raman_ is defined as follows:

$$N\mathrm{Raman}=\rho \times V\times {Ν}_{Α}/\mathrm{MW},$$

where *ρ* = 1.073 g/mL and MW = 110.18 g/mol are the density and molecular weight of benzene thiol and *V* is the collection volume of the liquid sample monitor. *N*_*A*_ = Avogadro’s number. *N*_SERS_ is defined as follows:

$$N_{\mathrm{SERS}}=\rho_{\mathrm{surf}}\times {Ν}_{Α}\times S_{\mathrm{surf}},$$

where *ρ*_surf_ is the surface coverage of benzene thiol on which has been reported as approximately 0.544 nmol/cm^2^, and *S*_surf_ is the surface area irradiated by exciting the laser.

To get an accurate and comparable estimation of the average enhancement factor, the Raman mode used for the calculation of the average EF must be selected carefully because the average EFs calculated from different Raman modes have a great deviation. For comparison, the three Raman modes associated with vibrations about the aromatic ring are presented in the inset of Figure 3a, and the average EFs of optimal substrate (CW300) which are calculated based on the intensities of the modes at 998/cm (C-H wag), 1,021/cm (C-C symmetric stretch), and 1,071/cm (C-C asymmetric stretch) are 2 × 10^8^, 5 × 10^8^, and 2 × 10^9^, respectively. However, while the average EFs calculated were based on the neat benzene thiol dependent on the choice of Raman mode strongly, the relative Raman enhancement between our SERS substrates (including the Klarite® substrate) were found to be relatively independent on the choice of Raman mode used for comparison, as shown in Figure 3a. Here, the intensities of the peak found at 998/cm, with the carbon-hydrogen wagging mode which is the furthest mode removed from the gold surface, were used to compute the average EFs. And the average EF of the Klarite® substrate was calculated to be 5.2 × 10^6^, which is reasonable because the enhancement factor for the inverted pyramid structure of Klarite® substrates relative to a non-enhancing surface is rated to have a lower bound of approximately 10^6^.

## Results and discussion

The average EFs for our SERS substrates were calculated and are presented in Figure 3b as a function of *d* (black open squares). For each substrate, more than 80 spectra were collected at various positions to ensure that a reproducible SERS response was attained. Spatial mapping with an area larger than 20 μm × 20 μm of the SERS intensity of CW300 was shown in Figure 3c as an example. It was certified that the relative standard deviation (RSD) in the SERS intensities were limited to approximately 30% within a given substrate, which is similar with the result of other groups [17]. The SERS response at a given point on the substrate was found to be highly reproducible, with variations in the detected response being limited to about 7%.

According to the results shown in Figure 3b, with the increase in *d*, when *d* ≤ 300 nm, the gap size *g* decreases, and the average EF increases. The highest average EF, 2 × 10^8^, is obtained when *d* = 300 nm. But when *d* ≥ 350 nm, the average EF decreases abruptly to about 5 × 10^5^. This is because a relatively continuous and rugged layer has formed on the top of the nanopillars and, consequently, the high density and deep nanogaps were covered up when *d* ≥ 350 nm.

Additionally, as shown in Figure 3a,b, the Raman intensity of the peak at 998/cm of our optimal SERS substrate (CW300) is about 200 times as large as that of the Klarite® substrate. But the calculated highest average EF of CW300, 2 × 10^8^, is only about 40 times as large as the average EF of the Klarite® substrate, 5.2 × 10^6^. This is because the surface area (*S*_surf_) of CW300 is about four times as large as the *S*_surf_ of the Klarite® substrate. The large surface area of our substrate is induced by the high density and large depth of the nanogap structure. In other words, the high density and large depth of the nanogap structure of our substrate provide dense strong 'hot spots’ and an enormous Raman intensity but yields a relative small average EF. As shown in Figure 3a, an obvious background signal is found in the Raman spectrum of the Klarite® substrate, which almost cannot be found in the Raman spectrum of our substrate. Manifestly, our high density and deep nanogap structure substrates have an advantage for application.

To gain a better understanding on the role of plasmonic coupling in the SERS effect, COMSOL calculations of the predicted SERS enhancement with the parameters estimated according to the SEM images were carried out and presented as a function of gap size in Figure 3d. All of the simulation values presented in Figure 3d are normalized to the calculated SERS enhancement (E^4^) for the structure of CW50. And the measured average EFs shown in Figure 3d are also normalized to the measured average EFs of the SERS substrate CW50. Our experimental results agree with the simulations, both showing a dramatic increase in the average EFs with the decrease in the gap size, which is believed to be caused by the plasmonic coupling from the neighboring nanopillars. As shown, the experimental average EFs are larger than the simulations, which is because of the neglect of the roughness of the nanopillar surface in the simulating.

## Conclusions

In conclusion, through a simple low-cost and high-output method-depositing Au film, we engineer the ordered array of nanopillars structure on the wing to form large-area high-performance SERS substrate. By this method, the gap size between the nanopillars is fine defined and SERS substrates with sub-10-nm gap size are obtained, which have the highest average EF of about 2 × 10^8^. The dramatic increase in the average EFs with the decrease in the gap size induced by the plasmonic coupling from the neighboring nanopillars is certified. In this work, the natural and low-cost cicada wings were used as the templates directly; so, our SERS substrates are environment-friendly. Our low-cost environment-friendly large-area uniform reproducible and ultra-sensitive SERS substrates have huge advantages for applications and theoretical studies.

## Abbreviations

SERS: Surface-enhanced Raman scattering; EF: Raman enhancement factor; EBL: Electron-beam lithography; FIBL: Focused ion-beam lithography; SAM: Self-assembled monolayer; SEM: Scanning electron microscope; RSD: Relative standard deviation.

## Competing interests

The authors declare that they have no competing interests.

## Authors’ contributions

QJ conceived of the study, carried out the fabrication of the SERS substrates, measurement, analysis, and simulation and drafted the manuscript. LY participated in the SERS spectra analysis and discussion. YM and WQ participated in the SEM measurements and SERS spectra measurements. CZ, WW, LW, and YX participated in the simulation. XJ and SQ are the PIs of the project and participated in the design of the study, revised the manuscript, and conducted the coordination. All authors read and approved the final manuscript.
